# First report of anti-*Toxoplasma gondii* antibodies in the sand-dune tuco-tuco (*Ctenomys flamarioni*) from southern Brazil

**DOI:** 10.1590/S1984-29612026040

**Published:** 2026-09-11

**Authors:** Matheus Guzzi Rubini, Aluizio Araújo Passos, Gabrieli Zanette, Greice Zorzato Gonchoroski, Thales Renato Ochotorena de Freitas, Daniel Galiano

**Affiliations:** 1 Universidade Federal da Fronteira Sul - UFFS, Laboratório de Ecologia e Conservação, Erechim, RS, Brasil; 2 Universidade Federal do Rio Grande do Sul - UFRGS, Departamento de Genética, Laboratório de Citogenética e Evolução, Porto Alegre, RS, Brasil

**Keywords:** Protozoans, Rodentia, subterranean rodents, tuco-tucos, Protozoários, Rodentia, roedores subterrâneos, tuco-tucos

## Abstract

The subterranean rodent *Ctenomys flamarioni* is an endemic species threatened by the proximity to anthropogenic environments, a factor that increases its contact with domestic animals. The protozoan *Toxoplasma gondii* is of particular concern because its definitive hosts include domestic and wild felids, while several species of wild mammals act as intermediate hosts. The present study aimed to evaluate the seroprevalence of *T. gondii* in two populations of *C. flamarioni* in coastal dune areas of southern Brazil with different degrees of anthropogenic disturbance. A total of 17 individuals were captured using Oneida Victor traps, including seven from a low anthropization area and ten from a highly anthropized area. In the field, blood samples were collected from the cranial vena cava, and the animals were released at their capture site. Serum samples were analyzed using an indirect hemagglutination assay. The overall seroprevalence was 41.2%, with similar values between areas: 42.9% (low anthropization) and 40.0% (highly anthropized). Our results demonstrate that *C. flamarioni* is exposed to *T. gondii* in environments with both low and high degrees of anthropogenic influence. The detection of this zoonotic parasite highlights the role of human-altered environments in facilitating pathogen transmission to wildlife.

## Resumo

O roedor subterrâneo *Ctenomys flamarioni* é uma espécie endêmica ameaçada pela proximidade com ambientes antropizados, fator que aumenta seu contato com animais domésticos. O protozoário *Toxoplasma gondii* é motivo de preocupação particular, uma vez que seus hospedeiros definitivos incluem felídeos domésticos e silvestres, enquanto diversas espécies de mamíferos silvestres atuam como hospedeiros intermediários. O presente estudo teve como objetivo avaliar a soroprevalência de *T. gondii* em duas populações de *C. flamarioni* em áreas de dunas costeiras do sul do Brasil, com diferentes graus de perturbação antrópica. Um total de 17 indivíduos foi capturado utilizando armadilhas Oneida Victor, sendo sete provenientes de uma área com baixa antropização e dez de uma área altamente antropizada. Em campo, amostras de sangue foram coletadas da veia cava cranial, e os animais foram soltos no local de captura. As amostras de soro foram analisadas por meio de um ensaio de hemaglutinação indireta. A soroprevalência geral foi de 41.2%, com valores semelhantes entre as áreas: 42.9% (baixa antropização) e 40.0% (alta antropização). Nossos resultados demonstram que *C. flamarioni* está exposto ao *T. gondii* em ambientes com baixos e altos graus de influência antrópica. A detecção desse parasita zoonótico ressalta o papel dos ambientes alterados pelo homem em facilitar a transmissão de patógenos para a fauna silvestre.

## Introduction

The subterranean rodent *Ctenomys flamarioni* Travi, 1981, known as the sand-dune tuco-tuco, is endemic to southern Brazil, inhabiting the coastal dunes of the coastal plain of Rio Grande do Sul State (Freitas, 2016). The species is classified as Endangered by the International Union for Conservation of Nature (IUCN) (Freitas et al., 2019) and Vulnerable by the Instituto Chico Mendes de Conservação da Biodiversidade (ICMBio) (Freitas et al., 2024). *Ctenomys flamarioni* suffers from habitat degradation driven by urbanization, agricultural activities, domestic animals, coastal development, dune fragmentation, and *Pinus* spp. silviculture (Fernández-Stolz et al., 2007; Freitas et al., 2019). Among these threats, proximity to anthropogenic areas increases the risk of exposure to parasites transmitted by domestic animals, negatively impacting rodent populations through increased predation and disease exposure (Beineke et al., 2015; Echenique et al., 2018; Wright et al., 2022).

In this context, *Toxoplasma gondii* (Sarcocystidae) stands out as a potential threat to *Ctenomys flamarioni*, and is one of the most significant animal parasites. This parasite has a global distribution, affecting approximately 30% of the human population and infecting a wide range of endothermic animals (Frenkel, 2000; Montoya & Liesenfeld, 2004). According to a systematic review and meta-analysis of *T. gondii* seroprevalence in rodents, which evaluated 105 studies involving 26,221 rodents between 1970 and 2018, the estimated global seroprevalence was 6% (95% CI: 6–7%), with higher seroprevalence rates reported in Africa (24%) and South America (18%) (Galeh et al., 2020). Other studies have investigated the seroprevalence of *T. gondii* in wild marsupials and rodents in different regions of the Atlantic Forest. Siqueira et al. (2013) investigated the presence of antibodies against *T. gondii* in 174 wild rodents captured in Atlantic Forest fragments and urban areas of Pernambuco, and found that 5.7% (10) tested positive for antibodies by the modified agglutination test. Gennari et al. (2015) evaluated samples from 151 rodents from the Atlantic Forest in São Paulo State and found that 8.6% (13/151) were seropositive using the modified agglutination test. Furthermore, using the same test, Horta et al. (2018) reported that 5.3% (4/75) and 3.3% (2/60) of small mammals tested positive for anti-*T. gondii* IgG antibodies in two regions of the Caatinga biome. Regarding subterranean rodents of the genus *Ctenomys*, no published data for Brazilian species are currently available.

The life cycle of *T. gondii* is inextricably linked to domestic and wild felids, which act as definitive hosts, excreting viable oocysts in their feces. Transmission can occur via the fecal–oral route, as well as through consumption of infected meat, and by transplacental transmission from mother to fetus (Hill et al., 2005). In intermediate hosts, such as rodents, infection can cause clinical manifestations including encephalomyelitis, cardiomyopathy, and tissue lesions, ultimately resulting in mortality (Shapiro et al., 2019). Considering the interaction among domestic animals, humans, and wildlife, understanding the dynamics of infection in natural populations is critical, as small rodents can serve as reservoirs and sources of infection for felines and humans (Galeh et al., 2020). Although the impact of domestic parasites on wildlife is becoming increasingly documented (Echenique et al., 2018), studies on wild rodents remain predominantly restricted to regions outside of Brazil, highlighting the need for investigations involving native species. In this context, the present study aimed to evaluate the seroprevalence of *Toxoplasma gondii* in populations of *Ctenomys flamarioni* and to record the presence of domestic dogs and cats in coastal dune areas of southern Brazil with different degrees of anthropogenic disturbance, thereby providing information relevant to the conservation of this species.

## Material and Methods

This study evaluated two populations of *C. flamarioni* located in the coastal dune region of Rio Grande do Sul State. Animal sampling was conducted in March 2025 at two natural sites: 1) an area with a high degree of anthropogenic disturbance, corresponding to a population located on the northern coast, in the municipality of Xangri-lá, RS (29°48'03"S, 50°02'37"W), characterized by intense tourist activity, extensive urbanization, and the constant presence of domestic animals in the dunes; and 2) an area with a low degree of anthropogenic disturbance, corresponding to a population located on the southern coast, in the municipality of Mostardas, RS (31º 06' 25"S, 50º55'16"W), characterized by little to no tourist activity and a preserved environment without visible anthropogenic impacts. The animals were live-captured using Oneida Victor No. 0 traps with rubber covers, to avoid injuries of captured animals. These traps were placed at the entrance of burrows, identified by the presence of freshly excavated sand and sand mounds, and were checked every 15 minutes during the sampling period. After capture, the animals were kept in a holding box until the collection of biological material (blood). The animals were weighed and subsequently anesthetized with ketamine (100 mg/mL) according to the protocol described by Stein et al. (2010). To collect blood samples, 0.5 mL micro syringes were used. In the field, blood samples were collected from the cranial vena cava, transferred to tubes without anticoagulants, and the animals were returned to the holding box until full recovery before being immediately released at the capture site. Blood samples were stored at -20°C until processing. In the laboratory, the blood was centrifuged to obtain the serum and stored at -20°C. To assess the exposure of *C. flamarioni* to *T. gondii*, serum samples were subjected to an indirect hemagglutination assay (Toxotest HAI, Wiener kit). A dilution of 1:16 was adopted as the cutoff point and considered positive, according to the manufacturer's instructions. The seroprevalence of *T. gondii* was calculated as the ratio between positive animals and the total number of individuals captured in each area.

Additionally, during each sampling day, the presence of domestic animals (cats and dogs) was qualitatively assessed at the same trapping sites used for *C. flamarioni* sampling. Throughout the fieldwork, observers visually recorded the direct presence of cats and dogs, as well as indirect evidence of their presence, such as feces and signs of activity in the dune environment.

## Results and Discussion

Seventeen individuals of *Ctenomys flamarioni* were sampled (9 males and 8 females), including seven captured in the area with low anthropogenic disturbance, and ten in the area with high anthropogenic disturbance (Table 1). The overall seroprevalence was 41.2% (7/17). Seropositivity rates were similar between areas, reaching 42.9% (3/7) in the site with low anthropogenic disturbance and 40.0% (4/10) in the highly disturbed site (Figure 1). In the area of low disturbance, only two domestic dogs were occasionally observed during the sampling period, although indirect evidence of domestic animal activity—including tracks, feces, and digging marks—was also recorded. In contrast, the area with high anthropogenic disturbance was characterized by frequent human activity, including people accompanied by dogs, more than 20 free-ranging dogs in the dunes, numerous signs of domestic animal activity (e.g., tracks, feces, and dog digging marks), and domestic cats associated with nearby residences.

**Table 1:** Collected samples, sex, weight, and dilutions for the presence of anti-*Toxoplasma gondii* antibodies in the sand-dune tuco-tuco (*Ctenomys flamarioni*) from southern Brazil. Serum samples were subjected to the indirect hemagglutination assay (Toxotest HAI, Wiener kit).

**Area**	**Samples**	**Sex (M/F)**	**Weight (g)**	**Dilution**
Low degree of anthropization (Mostardas/RS)	1	F	240	-
	2	F	110	-
	3	F	300	-
	4	M	300	1:16*
	5	F	250	1:32*
	6	M	115	-
	7	M	320	1:16*
High degree of anthropization (Xangri-lá/RS)	8	M	190	-
	9	M	260	1:16*
	10	F	240	1:16*
	11	F	250	1:8
	12	M	260	1:16*
	13	F	240	1:8
	14	F	220	1:8
	15	M	305	1:16*
	16	M	220	1:8
	17	M	410	-

The 1:16 dilution was adopted as the cutoff point and considered positive (*). The dash (-) indicates that no agglutination was observed at the initial dilution.

**Figure 1:**
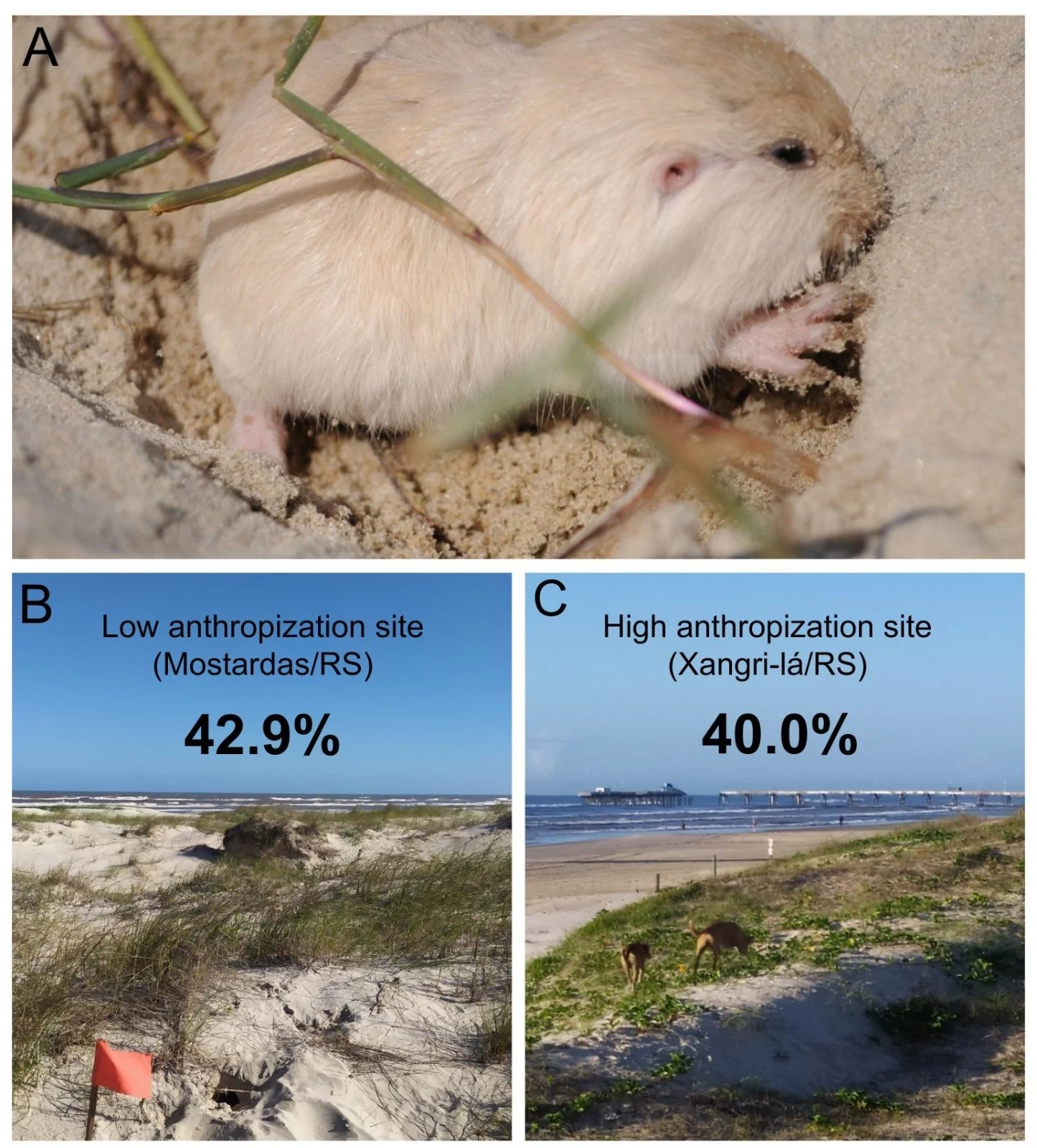
Results of seroprevalence of *T. gondii* in individuals of the species *Ctenomys flamarioni* (A) in environments with different degrees of anthropization (B - Low; C - High). Serum samples were subjected to the indirect hemagglutination assay (Toxotest HAI, Wiener kit).

Unexpectedly, the area with a low degree of anthropogenic disturbance showed a higher seroprevalence of *T. gondii*. Despite its relative isolation, this area showed evidence of domestic animals, indicating that interactions among wild animals, domestic animals, and humans occur even in remote locations. Previous studies on free-ranging rodents in Brazil have generally reported *T. gondii* seroprevalence ranging from 3.3% to 8.6%, including 5.7% in the Atlantic Forest of Pernambuco (Siqueira et al., 2013), 8.6% in the Atlantic Forest of São Paulo (Gennari et al., 2015), and 3.3% and 5.3% in two regions of the Caatinga biome (Horta et al., 2018). Brito et al. (2020) suggested that the higher seroprevalence observed in rodents from an agroforestry system (17.39%), compared with Atlantic Forest fragments (3.25%), resulted from greater anthropogenic pressure, increased environmental degradation, and reduced biodiversity, which likely intensified interactions between wild rodents, humans, and domestic animals. However, despite evaluating populations inhabiting areas with contrasting degrees of anthropogenic disturbance, the overall seroprevalence of *T. gondii* in *C. flamarioni* (41.2%) was markedly higher than those previously reported for free-ranging rodents in Brazil. Afonso et al. (2007) reported a higher seroprevalence of *T. gondii* in fossorial mammals inhabiting grasslands than in surface-dwelling small mammals from forest habitats in France. The authors attributed this difference to the fossorial lifestyle of these species, which results in frequent contact with soil potentially contaminated with oocysts during burrow excavation and foraging. This mechanism may also explain the high seroprevalence observed in *C. flamarioni* in the present study, suggesting that its fossorial habits increase the likelihood of exposure to environmentally dispersed oocysts.

Our results demonstrate that *C. flamarioni* is exposed to *T. gondii* in environments with both low and high degrees of anthropogenic influence, with a slightly higher seroprevalence in the less impacted area. The detection of this zoonotic parasite highlights the role of human-altered environments in facilitating pathogen transmission to wildlife. Although the epidemiological role of this species remains unclear, studies of zoonotic pathogens in wild populations are essential as indicators of environmental contamination and potential risks to biodiversity and human health. This study represents the first report of anti-*T*. *gondii* antibodies in *Ctenomys flamarioni*, expanding our knowledge of parasite occurrence within the genus *Ctenomys* and among subterranean mammals.

## Data Availability

All data used for the development of this study are available in the manuscript.
